# Effect of Gosha-Jinki-Gan on Levels of Specific mRNA Transcripts in Mouse Testes after Busulfan Treatment

**DOI:** 10.3390/biomedicines8100432

**Published:** 2020-10-19

**Authors:** Ning Qu, Kenta Nagahori, Miyuki Kuramasu, Yuki Ogawa, Kaori Suyama, Shogo Hayashi, Kou Sakabe, Masahiro Itoh

**Affiliations:** 1 Department of Anatomy, Division of Basic Medical Science, Tokai University School of Medicine, Kanagawa 259-1193, Japan; suyama@is.icc.u-tokai.ac.jp (K.S.); sho5-884@umin.ac.jp (S.H.); sakabek@tokai-u.jp (K.S.); 2 Department of Anatomy, Tokyo Medical University, Tokyo 160-8402, Japan; kenta-n@tokyo-med.ac.jp (K.N.); kitaoka@tokyo-med.ac.jp (M.K.); yogawa@tokyo-med.ac.jp (Y.O.); itomasa@tokyo-med.ac.jp (M.I.)

**Keywords:** oriental medicine, aspermatogenesis, specific mRNA transcript, anticancer treatment

## Abstract

With the increase in survival rates of cancer patients in recent years, infertility caused by anticancer treatments has become a significant concern for cancer survivors. Some studies have suggested that Sertoli cells play a key role in mediating testicular immunology in busulfan-induced aspermatogenesis. We recently demonstrated that Gosha-jinki-gan (TJ107), a traditional Japanese medicine, can completely recover injured spermatogenesis in mice 60 days after busulfan injection. In the present study, we sought to examine the levels of mRNA transcripts encoding markers of 25 Sertoli cell-specific products and 10 markers of germ cell differentiation. Our results demonstrated that only supplementation of TJ107 at day 60 after busulfan injection could significantly recover the increase in five mRNA species (*Amh*, *Clu*, *Shbg*, *Testin*, and *Il1a*) and the decrease in four mRNA species (*Aqp8*, *CST9*, *Wnt5a*, and *Tjp1*) in response to Busulfan (BSF) at day 120, with the increase of all examined spermatogenic markers.

## 1. Introduction

Busulfan (BSF; 1,4-butanediol dimethanesulfonate) is used as both a chemotherapeutic drug in the treatment of various malignancies [1,2] and an immunosuppressive drug in hematopoietic stem cell transplantation [1]. BSF treatment has been extensively shown to disrupt spermatogenesis by damaging germ cells and Sertoli cells [3,4]. Previous studies demonstrated that BSF may induce germ cell apoptosis through loss of c-kit signaling in a Fas/FasL- and p53-independent manner [5]. In BSF-induced apoptosis, p53 is a key protein through reactive oxygen species (ROS)-dependent activation of the extracellular signal-regulated kinase/p38 pathway, and decreased concentrations of deacetylated p53 result in spermatogonial cell resistance to apoptosis [6]. The in vivo and in vitro study showed that spermidine/spermine N1-acetyltransferase 2 (Sat2) is present in testicular Sertoli cells and that its expression is significantly increased by BSF treatment. Furthermore, upregulation of Sat2 by BSF alters the growth and function of Sertoli cells and causes male infertility [7]. Additionally, it has been demonstrated that BSF-induced spermatogenic cell damage upregulates tumor necrosis factor α and macrophage chemotactic protein 1 expression in Sertoli cells and facilitates macrophage infiltration into the testes [8]. These damaged germ cells in BSF-treated mice release endogenous Toll-like receptor (TLR) ligands to activate *TLR2* and *TLR4* in Sertoli cells, thus initiating endogenous inflammation in the testes [8,9].

The side effects of BSF include gonadal dysfunction amenorrhea and azoospermia in humans [10,11,12], and information on therapy for BSF-induced male infertility is limited. In mice, we previously found testicular weight loss, seminiferous tubule atrophy, spermatogenic cell degeneration, and apoptosis 60 days after BSF treatment (40 mg/kg) [13]. As a result of the limited information on therapy for BSF-induced male infertility, we attempted to determine the effects of several oriental Japanese medicines, such as Gosha-jinki-gan, Hachimi-jio-gan, and Hochu-ekki-to, on BSF-induced infertility [9,14]. We demonstrated that Gosha-jinki-gan (TJ107) was able to completely normalize testicular immunopathology and to promote recovery from severe aspermatogenesis following BSF treatment in mice [9]. Male 4-week-old C57BL/6j mice were administered a single intraperitoneal injection of BSF and were then fed a normal diet for 60 days and then a TJ107 or TJ107-free (normal) diet for another 60 days. After BSF treatment, the epididymal sperm count progressively decreased from 60 days (1.575 ± 0.308 × 10^5^ cells) to 120 days (0.217 ± 0.019 × 10^5^ cells) in the normal diet group. In the T107 diet group, dramatic recovery of these variables (21.680 ± 1.700 × 10^5^ cells) was observed at 120 days, which was similar to the results of normal mice (20.500 ± 2.462 × 10^5^ cells) [9]. In the current study, we examined whether TJ107 could recover spermatogenesis at 120 days following administration with BSF. We further examined the markers of Sertoli cell-specific products and germ cell differentiation in order to clarify the mechanism of the different therapeutic effects of TJ107 at different times of administration on BSF-induced infertility.

## 2. Materials and Methods

### 2.1. Animals

C57BL/6J male mice at 4 weeks of age (weighing 16 g–18 g) were purchased from SLC (Shizuoka, Japan) and were kept in the Laboratory Animal Center of Tokyo Medical University (Tokyo Medical University Animal Committee; no. S27001-01042016 (1 April 2015) and no. H28016-01042017 (1 April 2016)) and the Animal Laboratory of Support Center for Medical Research and Education, Tokai University (Tokai University Animal Committee; no. 171058-01042017 (1 April 2017)). The mice were maintained at a temperature of 22 °C–24 °C and a relative humidity of 50%–60%, with a 12-h light–dark cycle.

### 2.2. Preparation of a Gosha-Jinki-Gan Diet

Gosha-jinki-gan (TJ107) (extract granules in powdered form; no. 2120107030) was manufactured by Tsumura & Co. (Tokyo, Japan) according to Japanese and international manufacturing guidelines. TJ107 is a standardized prescription medicine containing 10 crude components: Rehmanniae radix, 5 g; Achyranthis radix, 3 g; Corni fructus, 3 g; Dioscoreae rhizome, 3 g; Plantaginis semen, 3 g; Alismatis rhizome, 3 g; Hoelen, 3 g; Moutan cortex, 3 g; Cinnamomi cortex, 1 g; and processed Aconite tuber, 1 g. A TJ107 diet was prepared as a mouse diet (standard diet; 23.1% crude protein (*w/w*), 5.1% crude fat, 5.8% crude ash, 2.8% crude fiber, and 55.3% nitrogen-free extract and mineral mixture; Oriental Yeast Co., Ltd., Tokyo, Japan) containing 5.4% (*w/w*) TJ107 extract.

### 2.3. Experimental Design

BSF (Sigma, St. Louis, MO, USA), 40 mg, was first dissolved in 1 mL dimethyl sulfoxide (DMSO; Sigma). The mice were weighed and given a single injection of BSF (16 μL–18 μL) diluted in 100 μL distilled water to achieve a final concentration of 40 mg per kg body weight.

The study mice were randomly divided into the following five groups: group I: control group (*n* = 15), mice that received a single intraperitoneal injection of DMSO and were fed the standard diet for 120 days after injection; group II: control + TJ107 group (*n* = 15), mice that received a single intraperitoneal injection of DMSO and were fed the TJ107 diet for 120 days after injection; group III: BSF group (*n* = 15), mice that received a single intraperitoneal injection of BSF and were fed the standard diet for 120 days after injection; group IV: BSF day 0 + TJ107 group (*n* = 15), mice that received a single intraperitoneal injection of BSF and were fed the TJ107 diet for 120 days after injection; and group V: BSF day 60 + TJ107 group (*n* = 15), mice that received a single intraperitoneal injection of BSF and were fed the standard diet for 60 days after injection and then the TJ107 diet for the remaining 60 days, until 120 days after injection. The general condition, food intake, and body weight were documented for all mice at 10-day intervals from 60 days to 120 days after injection. The experimental study protocols were performed in accordance with the guidelines of the National Institutes of Health and were approved by the Tokyo Medical University Animal Committee (no. S27001 and no. H28016) and the Tokai University Animal Committee (No. 171058).

At 120 days after injection, to assess in vivo fertilization ability, we cross-mated mice from each group (*n* = 5) with normal C57BL/6j female mice (8–10 weeks of age; SLC, Shizuoka, Japan) at a male-to-female ratio of 1:2 in a cage for 1 month and determined the fertility rate for each group.

To examine the effect of TJ107 with BSF on spermatogenesis, the remaining mice from each group (*n* = 5) were deeply anesthetized with pentobarbital (65 mg/kg body weight), the testes were removed for gravimetry, and epididymides were immediately removed to perform the epididymal spermatozoa count 120 days after injection. Furthermore, to clarify the causality of partially recovered spermatogenesis 60 days after injection in group I, group III, and group IV, mice (*n* = 5) were deeply anesthetized and the testes and epididymides were removed for examination 60 days after injection.

### 2.4. Histological Examination of the Testes

The testes of mice from each group (*n* = 5) were fixed whole with Bouin’s solution and embedded in plastic (Technovit7100; Kulzer & Co., Wehrheim, Germany). Sections (5 μm) were obtained at 15–20-μm intervals and were stained with Gill’s hematoxylin and 2% eosin Y (Muto PC, Tokyo, Japan) for observation under light microscope.

### 2.5. Analysis of the Specific mRNA Species in Testes Using Real-Time RT-PCR

The testes from mice in each group (*n* = 5) were examined. Total RNA was purified from each fresh tissue sample using TRIZOL reagent (Invitrogen Corp., Carlabad, CA) according to the manufacturer’s protocol, and its concentration was calculated from the extinction at 260 nm, as determined spectrophotometrically. For cDNA synthesis, 5 μg of total RNA was reverse-transcribed with a High Capacity cDNA Archive Kit (PE Applied Biosystems, Foster City, CA, USA) according to the standard protocol carried out with an iCycler Thermal Cycler (Bio-Rad, Hercules, CA, USA). The mixtures were stored at −80 °C until analysis. Real-time RT-PCR was performed on 2.5-μg cDNA using the validated SYBR Green gene expression assay in combination with SYBR Premix Ex Taq^TM^ (TaKaRa, Bio Inc., Ohtsu, Japan) for the 10 germ cell genes (*Stra8, Spo11, Tnp1, cKit, Gfra1, Vasa, Boll, Crem, Prm1,* and *Acrosin*), 25 Sertoli cell genes (*Amh, Aqp8, Ccnd2, Clu, Cldn11, Cst12, Cst9, Dhh, Espn, Fshr, Fyn, GATA1, Il1a, Inhba, Inhbb, Msi1, Rhox5, Testin, Shbg, Spata2, Sox9, Tjp1, Trf, Wt1,* and *Wnt5a*), and *GAPDH* as an internal control, respectively, with forward and reverse primers described previously [15,16,17,18,19] and was synthesized from Hokkaido System Science Co., Ltd. (Sapporo City, Hokkaido, Japan). Quantitative real-time PCR was performed in duplicate with the Thermal Cycler Dice Real-time System TP800 (TaKaRa), and the thermal profile used for amplification was 95 °C for 30 s followed by 40 cycles of 95 °C for 10 s, 60 °C for 10 s, and 72 °C for 10 s. The Thermal Cycler Dice Real-time System software (TaKaRa) was used to analyze the data, and the comparative C_t_ method (2∆∆*C*_t_) was used to quantify the gene expression levels. The results are expressed relative to *GAPDH*. The relative mRNA intensity was calculated, and the expression in the control group for each point was normalized to 1. The data are presented as mean ± standard deviation. Primers used in this analysis are shown in Table 1.

### 2.6. Epididymal Spermatozoa Count

Epididymal spermatozoa were recovered from both epididymes of mice in each group (*n* = 5). Briefly, the epididymides were dissected out and cut into six pieces in phosphate-buffered saline (PBS). All pieces were gently pipetted and then passed through a stainless-steel mesh. The epididymal spermatozoa were harvested by centrifugation at 400× *g* for 10 min and resuspended in 5 mL of PBS after washing three times with PBS.

### 2.7. Statistical Analysis

ANOVA and Tukey–Kramer post hoc test were used to analyze the differences between the multiple groups. A *p*-value < 0.05 was considered statistically significant.

## 3. Results

### 3.1. Supplemented TJ107 Significantly Recovered Spermatogenesis and Levels of mRNA Transcripts Encoding Markers of Germ Cell Differentiation in the BSF-Treated Group V Mice at Day 120

Following BSF treatment, the mean body weight of the mice in the BSF-treated groups (groups III, IV, and V) were significantly lower than that of control mice (group I) at the end of the experiment (day 120), regardless of TJ107 supplementation (Table 2). In contrast, only the group V mice showed significant and almost complete recovery of testicular weight, epididymal spermatozoa counts, and fertility rate to the levels found in group I (Table 2). The mice in group IV also showed significant recovery in their testicular weights, spermatozoa count, and fertility rate compared to the mice in group III, although they did not return to the control levels in group I (Table 2).

**Figure 1:**
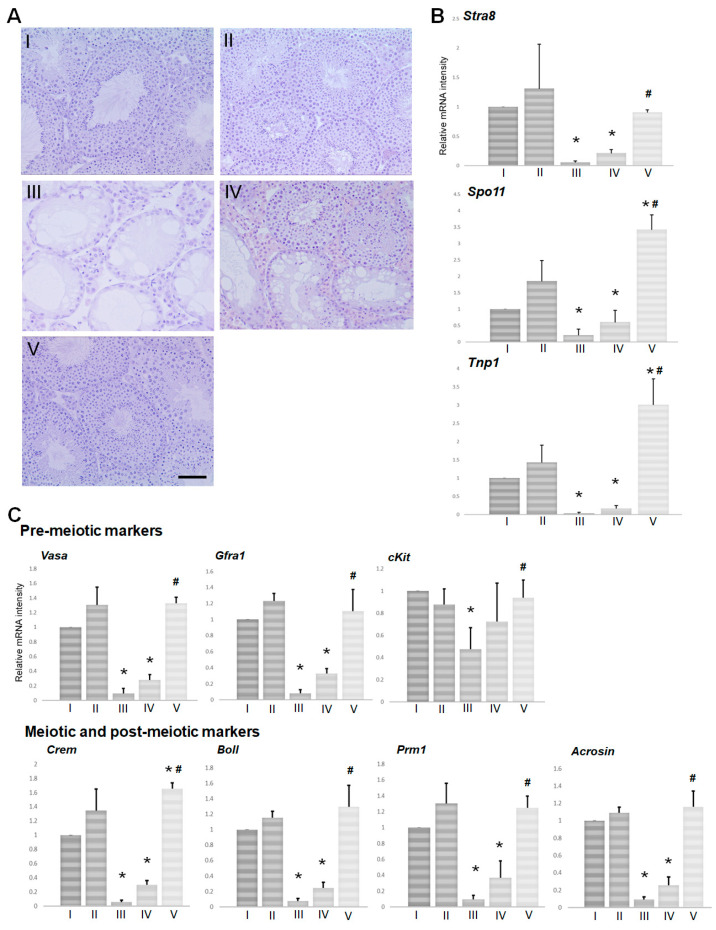
Testicular histology and the levels of three mRNA transcripts encoding markers of germ cell differentiation in each group at day 120 (*n* = 5): (**A**) testes sections show morphology in groups I–V. Intact seminiferous tubules showing normal germinal epithelium from the spermatogonia to spermatozoa are observed in groups I and II. Atrophic seminiferous tubules with azoospermia are observed in group III. The presence of both atrophic and intact seminiferous tubules with spermatogenesis was observed in the group IV mice. Normal-appearing seminiferous tubules are observed in the group V mice (bar = 50 µm). (**B**) Expression was measured by real-time RT PCR, and the results are expressed relative to the internal control *GAPDH*. Data show expression of the spermatogonial marker *Stra8*, the spermatocyte marker *Spo11*, and the spermatid marker *Tnp1*. The results are expressed as the mean values ± standard deviation of five mice in each group, and the y-axis shows relative mRNA intensity. (**C**) Expression was measured by real-time RT PCR, and the results are expressed relative to the internal control *GAPDH*. Data show expression of the premeiotic markers (*cKit, Gfra1,* and *Vasa*), and the meiotic and postmeiotic markers (*Boll, Crem, Prm1,* and *Acrosin*). The results are expressed as the mean values ± standard deviation of five mice in each group, and the y-axis shows relative mRNA intensity. * *p* < 0.05 vs. group I; ^#^ *p* < 0.05 vs. group III.

In groups I and II (no BSF treatment), histological examination of the testes demonstrated intact seminiferous tubules with normal spermatogenesis from spermatogonia to spermatozoa in the cycle of germinal epithelium (Figure 1A(I,II)). BSF treatment induced the appearance of atrophic seminiferous tubules and azoospermia in group III mice (Figure 1A(III)). A partial recovery of spermatogenesis was observed in group IV mice that received a TJ107 diet from day 0. Furthermore, in these mice, seminiferous tubules that exhibited intact spermatogenesis were found adjacent to the seminiferous tubules that showed aspermatogenesis as Sertoli cells only (Figure 1A(IV)). In contrast, in group V, the administration TJ107 from day 60 restored normal spermatogenesis in all the seminiferous tubules (Figure 1A(V)).

Furthermore, we compared the expression levels of mRNA species encoding premeiotic cells (*cKit, Gfra1,* and *Vasa*), meiotic and postmeiotic cells (*Boll, Crem, Prm1,* and *Acrosin*) (Figure 1C), the spermatogonial marker *Stra8*, the spermatocyte marker of *Spo11,* and the spermatid marker *Tnp1* (Figure 1B) [15,16,17,18,19,20,21,22,23] by real-time RT-PCR analysis in each group at day 120. The levels of all examined ten spermatogenesis markers were significantly reduced in group III but completely recovered in group V; the expression recovered slightly in group IV but remained significantly less than that in group V and normal mice.

### 3.2. Effect of TJ107 on Levels of mRNA Transcripts Encoding Markers of Sertoli Cell-Specific Products at Day 120

**Figure 2:**
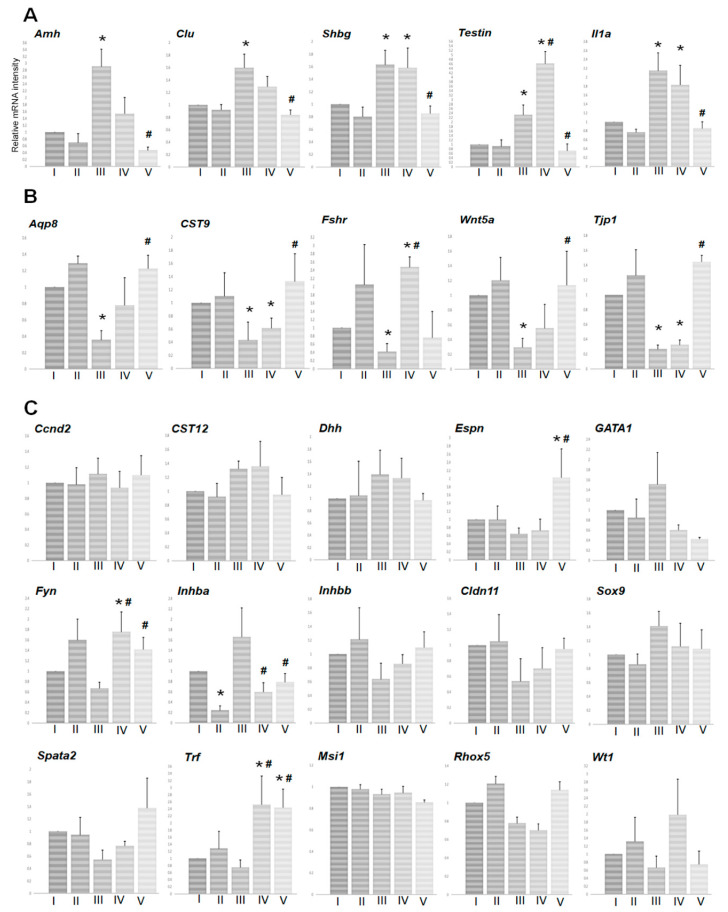
Effect of TL107 on the levels of 25 mRNA transcripts encoding markers of Sertoli cell-specific products in each group at day 120: expression was measured by real-time RT PCR, and the results are expressed relative to the internal control *GAPDH*. The results are expressed as the mean values ± standard deviation of five mice in each group, and the y-axis shows relative mRNA intensity. Transcripts showing a significant increase in levels after BSF treatment compared to control values are grouped in (**A**); transcripts showing a significant decrease in levels after BSF treatment compared to control values are grouped in (**B**). Transcripts that showed no change in levels after BSF treatment are grouped in (**C**). * *p* < 0.05 vs. group I; ^#^ *p* < 0.05 vs. group III.

To examine the effects of TJ107 on Sertoli cell function regulated by BSF-induced germ cell depletion, the levels of 25 Sertoli cell-specific mRNA species were measured in each group at day 120 (Figure 2). Five mRNA species (Amh, Clu, Shbg, Testin, and Il1a) showed significantly increased expression (Figure 2A) in response to BSF, and five mRNA species (Aqp8, CST9, Fshr, Wnt5a, and Tjp1) showed decreased expression (Figure 2B). Of these, Amh and Fshr showed a degree of recovered expression in group IV, while all ten mRNA species showed completely recovered expression in group V (Figure 2A,B). There were no significant changes in the expression levels of the remaining 15 mRNA species (Ccnd2, Cst12, Dhh, Espn, Fyn, GATA1, Inhba, Inhbb, Cldn11, Msi1, Rhox5, Spata2, Sox9, Trf, and Wt1) after BSF treatment in group III, although Fyn and Trf were markedly increased by TJ107 administration in groups II, IV, and V (Figure 2C).

### 3.3. Effects of Supplemented TJ107 from Day 0 on Spermatogenesis in Group IV at Day 60

**Figure 3:**
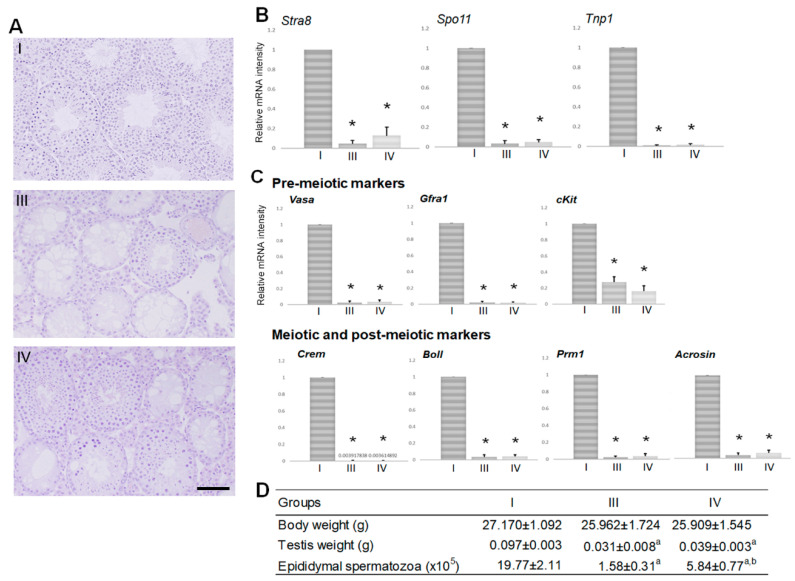
Testicular histology and levels of three mRNA transcripts encoding markers of germ cell differentiation in groups I, III, and IV at day 60 (*n* = 5): (A) seminiferous tubules showing normal spermatogenesis from spermatogonia to spermatozoa are observed in the group I mice. Both atrophic and intact seminiferous tubules with spermatogenesis were observed in the group III and group IV mice (bar = 40 µm). (B,C) Expression was measured by real-time RT PCR, and the results are expressed relative to the internal control *GAPDH*. (B) Data show expression of the spermatogonial marker *Stra8*, the spermatocyte marker *Spo11*, and the spermatid marker *Tnp1*. (C) Data show expression of the premeiotic markers (*cKit, Gfra1,* and *Vasa*), and the meiotic and postmeiotic markers (*Boll, Crem, Prm1,* and *Acrosin*). The results are expressed as the mean values ± standard deviation of five mice in each group, and the y-axis shows relative mRNA intensity. * *p* < 0.05 vs. group I. (D) Testicular weights and epididymal spermatozoa count in groups I, III, and IV. Data are presented as mean ± standard deviation. ^a^ *p* < 0.05 vs. group I; ^b^ *p* < 0.05 vs. group III.

We performed histological analysis and analysis of the specific mRNA species in the testes of group III and IV mice at day 60 in order to clarify the mechanism of the different therapeutic effects of TJ107 at different administration times on BSF-induced aspermatogenesis. The body weight of the mice in groups III and IV were not significantly different to group I mice (treated with DMSO) at day 60 (Figure 3D). In group IV mice, the epididymal spermatozoa counts, but not the testicular weights, were significantly increased compared to group III mice but still significantly lower compared to group I mice (Figure 3D). In the histological examination of the testes, spermatogenesis from spermatogonia to spermatozoa in the cycle of germinal epithelium were observed in the seminiferous tubules of normal group I mice (Figure 3A(I)). In contrast, in the BSF-treated mice without (group III) or with (group IV) a TJ107 diet, both atrophic seminiferous tubules and intact seminiferous tubules with spermatogenesis was observed (Figure 3A(III,IV)). In the measurement of mRNA levels, spermatogenesis markers encoding pre-meiotic cells (*cKit*, *Gfra1*, and *Vasa*), meiotic and postmeiotic cells (*Boll*, *Crem*, *Prm1*, and *Acrosin*), the spermatogonial marker *Stra8*, the spermatocyte marker of *Spo11*, and the spermatid marker *Tnp1* were significantly decreased in groups III and IV compared to group I. Furthermore, the expressions of all ten mRNAs were not significantly different between groups III and IV (Figure 3B,C).

**Figure 4:**
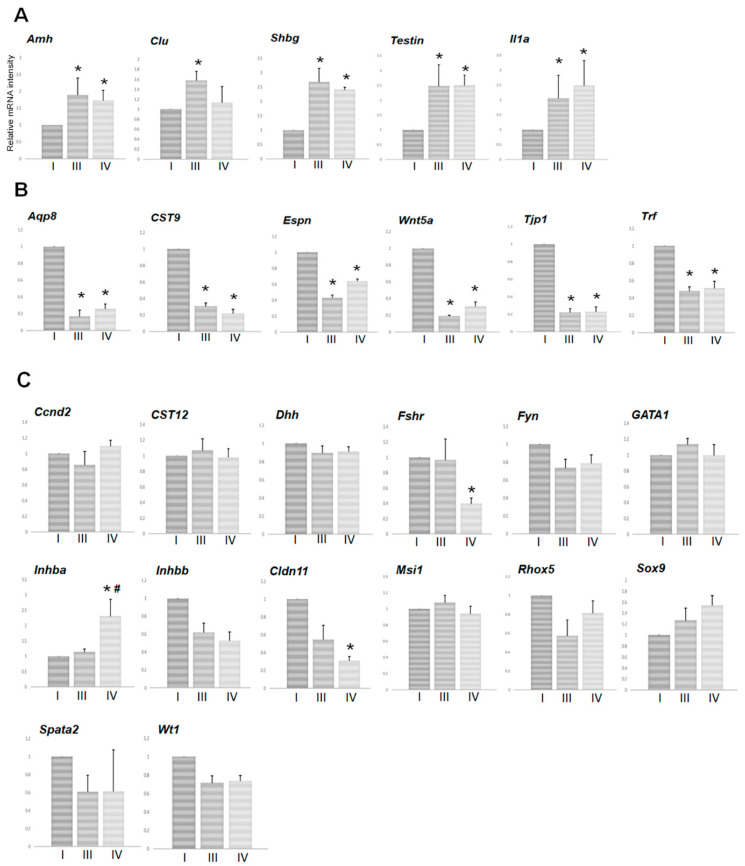
Effect of TL107 on the expression levels of 25 mRNA transcripts encoding markers of Sertoli cell-specific products in groups I, III, and IV mice at day 60: expression was measured by real-time RT PCR, and the results are expressed relative to the internal control *GAPDH*. The results are expressed as the mean values ± standard deviation of five mice in each group, and the y-axis shows relative mRNA intensity. Transcripts showing a significant increase in levels after BSF treatment compared to control values are grouped in (**A**); transcripts showing a significant decreased in levels after BSF treatment compared to control values are grouped in (**B**); and transcripts showing no change in levels after BSF treatment are grouped in (**C**). * *p* < 0.05 vs. group I; ^#^ *p* < 0.05 vs. group III.

Upon examining the effects of TJ107 on Sertoli cell function after BSF treatment, the same Sertoli cell-specific mRNA species levels were measured in groups III and IV at day 60 (Figure 4). In response to BSF at day 60, the levels of five mRNA species (*Amh*, *Clu*, *Shbg*, *Testin*, and *Il1a*) were significantly increased (Figure 4A), while the levels of six mRNA species (*Aqp8*, *CST9*, *Espn*, *Wnt5a*, *Tjp1*, and *Trf*) were significantly decreased (Figure 4B); among these, there were no significant differences between groups III and IV. There were no significant differences in the expression levels of the remaining 14 mRNA species (*Ccnd2*, *Cst12*, *Dhh*, *Fshr*, *Fyn*, *GATA1*, *Inhba*, *Inhbb*, *Cldn11*, *Msi1*, *Rhox5*, *Spata2*, *Sox9*, and *Wt1*) following BSF treatment in group III. However, *Fshr* and *Cldn11* were decreased and *Inhba* was increased in group IV mice following administration of TJ107 (Figure 4C).

## 4. Discussion

In this study, we examined the different effects of TJ107 on BSF-induced aspermatogenesis for 120 days in response to different times of administration after BSF injection. Our results demonstrated that only supplemented TJ107 at day 60 after BSF injection could significantly recover spermatogenesis with the development of the meiotic and postmeiotic stages from spermaotgonial cells at day 120. On further examination of the 25 markers of Sertoli cell-specific products, we demonstrated that only supplementation of TJ107 at day 60 after BSF injection could significantly recover the increase in five mRNA species (*Amh*, *Clu*, *Shbg*, *Testin*, and *Il1a*) and the decrease in four mRNA species (*Aqp8*, *CST9*, *Wnt5a*, and *Tjp1*) in response to BSF at day 120, with the increase of germ cell differentiation. To the best of our knowledge, this is the first study to demonstrate the effects of the oriental medicine TJ107 on Sertoli cells in the context of spermatogenesis. Furthermore, the abovementioned mRNA species showed the same changes in response to BSF at day 60; therefore, it is likely that these nine mRNA transcripts encoding Sertoli cell-specific products are associated with male infertility after BSF chemotherapy.

We have previously demonstrated that TJ107 or administration of Hachimi-jio-gan (TJ7) and Hochu-ekki-to (TJ41) in combination were able to completely normalize testicular immunopathology and to promote recovery from severe aspermatogenesis after BSF treatment in mice [9,14]. We further showed that BSF treatment upregulated the expression of *TNF-α*, *MCP-1*, *TLR2*, and *TLR4* in the testes, which was completely reversed by TJ107, or TJ7 and TJ41 coadministration [9,14]. This study is the first to examine whether TJ107 could recover spermatogenesis after 120 days by administering from the day of BSF injection. It has been previously demonstrated that treatment with BSF has no effect on the intratesticular levels of testosterone, which confirms the findings that germ cell ablation has no significant effect on testosterone levels [24,25]. Furthermore, O’Shaughnessy demonstrated that the levels of mRNA transcripts encoding Leydig cell-specific products related to steroidogenesis were unaffected by BSF treatment [23]. In the current study, we further examined the markers of Sertoli cell-specific products and germ cell differentiation in order to clarify the mechanism of the different therapeutic effects of TJ107 observed at different times of administration on BSF-induced infertility.

The mice treated with BSF showed a significant decrease in body weight, testes weight, and epididymal spermatozoa counts at day 60 (Figure 3A(III)), which was more pronounced at day 120 (Figure 1A(III)) in agreement with our previous studies [9,14]. Furthermore, supplementation with TJ107 could lead to significant improvement in body weight but not complete recovery in groups IV and V (Table 2). In contrast, complete recovery of testes weight, the development of spermatogonial cells to meiotic and postmeiotic stages including the generation of testicular spermatozoa, epididymal spermatozoa counts, and fertility rate was only observed in mice that subsequently received TJ107 at day 60 after BSF treatment in group V (Table 2; Figure 1A(V),B), while a modicum of improvements was detected in that of group IV (Table 2; Figure 1A(IV),B). Furthermore, the difference in the offspring number between group IV (0.20 ± 0.09) and V (3.27 ± 2.01) is also detected. These results indicated that only administration of TJ107 at day 60 after BSF treatment might able to completely regenerate the seminiferous epithelium that was injured by treatment with BSF.

We next examined the effects of TJ107 on Sertoli cell function after BSF treatment at day 60 and day 120 in order to clarify the mechanism of the different therapeutic effects. BSF treatment led to an increase in five mRNA species (*Amh*, *Clu*, *Shbg*, *Testin*, and *Il1a*) from day 60 to day 120, which were only recovered by supplementation with TJ107 in group V at day 120 (Figure 2A) but not in group IV at both day 60 (Figure 4A) and day 120 (Figure 2A). Furthermore, BSF treatment led to a decrease in mRNA species at day 60 *(Aqp8*, *CST9*, *Espn*, *Wnt5a*, *Tjp1*, and *Trf)* and day 120 (*Aqp8*, *CST9*, *Fshr*, *Wnt5a*, and *Tjp1*), and four mRNA species (*Aqp8*, *CST9*, *Wnt5a* and *Tjp1*) were recovered by supplementation with TJ107 in group V at day 120 (Figure 2B). The mRNAs that were decreased showed no recovery in group IV at day 60 (Figure 4B) and at day 120, with the exception of *Fshr,* which was significantly increased (Figure 2B). Furthermore, administration of TJ107 affected the expression of *Inhba* and *Cldn11* at day 60 (Figure 4C) and *Fyn* and *Inhba* at day 120 (Figure 2C), although these mRNA species showed no changes after BSF treatment. The mRNA species that were stably expressed in response to BSF (increased in five mRNA species: *Amh*, *Clu*, *Shbg*, *Testin*, and *Il1a*; decreased in four mRNA species: *Aqp8*, *Cst9*, *Wnt5a*, and *Tjp1*) showed restored expression and completely recovered spermatogenesis in group V mice.

Spermatogenesis is a complex and tightly regulated process that leads to continuous production of male gametes, the spermatozoa. The maintenance of spermatogonial stem cells provides the foundation for life-long spermatogenesis. In mice, *Wnt5a* is male-specifically upregulated within testicular interstitial cells at the onset of gonad differentiation and has been shown to be a cell-extrinsic factor that supports self-renewal of spermatogonial stem cells [26]. In the present study, the *Wnt5a* mRNA species decreased in response to BSF, with severe aspermatogenesis that was recovered by supplementation with TJ107 in group V mice. Sertoli cells are somatic cells of the testis that support the architectural stability of germ cells and conserve the microenvironment and blood–testis barrier. Moreover, Sertoli cells produce various hormones that play essential roles in gonadal development and the survival of spermatogonial cells. Anti-Müllerian hormone (*Amh*) is one such hormone expressed in Sertoli cells of the fetal and adult testes [27,28]. Furthermore, sex hormone-binding globulin (*Shbg*) is produced and secreted by the liver into the bloodstream, where it binds sex steroids and regulates their bioavailability. In rodents, the *Shbg* gene is also expressed in Sertoli cells and has been shown to significantly affect total-androgen levels [29]. In our study, the rise in *Amh* and *Shbg* following treatment with BSF may be consistent with regulation by the loss of germ cells. Throughout spermatogenesis, the Sertoli cell blood–testis barrier is an important structural component consisting of tight junction proteins such as occludin, Cldn11, and tight junction protein 1 (Tjp1) [30]. Furthermore, the Sertoli cell blood–testis barrier is strictly regulated by cytokines such as interleukin-1α (*Il1α*), and germ cells are known to control production of this cytokine by Sertoli cells [31]. Although we did not detect BSF-induced impairment of the blood–testis barrier [32], the decreased expression of *Tjp1* and the increased expression of *Il1α* warrant further investigation.

It is well known that *Testin* is a testosteron-responsive Sertoli cell secretory product. *Testin* is localized on the surface of Sertoli cells which allows contact with germ cells [33,34]; consequently, depletion of germ cells leads to an increase of *Testin* mRNA [34], which is consistent with the results of the present study. Some studies have demonstrated that clusterin (Clu) is secreted by the Sertoli cells in rats [35] and deposited onto the membranes of elongating spermatids and mature spermatozoa [36]. Recently, Fukuda et al. reported the use of the seminal *Clu* level as a biomarker for assessment of spermatogenic status in infertile men [37]. In the present study, we first demonstrated that *Clu* mRNA was increased in response to germ cell depletion by BSF treatment and was restored with completely recovered spermatogenesis.

Previous studies have shown various molecular mechanisms by which BSF mediates toxic effects on spermatogenesis [5,38]; these include targeting genes expressed in the testis. It is well known that Sertoli cells support and organize the stability of spermatogenesis and conserve the testicular microenvironment. In line with this, O’Shaughnessy et al. demonstrated that the transcript levels of nine mRNAs were increased (*Amh*, *Clu*, *Cldn11*, *Cst9*, *Cys12*, *Il1a*, *Shbg, Testin,* and *Wnt5a*) and two were decreased (*Spata2* and *Sympk*) in response to BSF treatment [23]. Furthermore, there were no changes in the expression levels of 16 Sertoli cell genes (*Ccnd2*, *Cldn11*, *Cst12*, *Dhh*, *Espn*, *Fshr*, *Fyn*, *GATA1*, *Inhba*, *Inhbb*, *Msi1*, *Rhox5*, *Spata2*, *Sox9*, *Trf*, and *Wt1*) in our present study. Among these Sertoli cell genes, 11 mRNAs (*Ccnd2*, *Dhh*, *Espn*, *Fshr*, *Fyn*, *GATA1*, *Inhba*, *Msi1*, *Rhox5*, *Sox9*, and *Wt1*) were reported by O’Shaughnessy et al. [23] and the different expressions were seen in 5 mRNAs (*Cldn11*, *Cst12*, *Inhbb*, *Spata2*, and *Trf*). The different dose of BSF (30 mg/kg), different age of recipient mice (15 weeks), and different experimental period (50 days after BSF injection) are causally related to the differences observed between the two studies. Furthermore, Sertoli cell activity is particularly sensitive to regulation by meiotic germ cells [39,40,41], and we demonstrate altered Sertoli cell function after BSF treatment at day 120, with intact spermatogenesis showing Sertoli cells only in the seminiferous tubules. Therefore, Sertoli cell factors may be intimately interrelated with spermatogenesis after BSF chemotherapy and may assist with providing an estimation of male fertility. Although the mechanism by which TJ107 led to improvement only 60 days after injection of BSF but not from the beginning for 120 days is not clear yet, we will sort out Sertoli cells before RNA isolation step and immune-stain testes sections with antibodies recognizing the analyzed markers to experimentally prove the fact of Sertoli cell-specific changes in gene expression upon BSF or BSF + TJ107 treatments in our next study. For possible future therapeutic strategies to male infertility treatment, mainly after chemotherapy, we will also examine the presence of germ cells by immunofluorescence-staining of each spermatogenic stage upon BSF or BSF + TJ107 treatments.

## Figures and Tables

**Table 1:** Primers used for real-time RT-PCR.

Genes	Forward Primer	Reverse Primer
*GAPDH*	TGTGTCCGTCGTGGATCTGA	TTGCTGTTGAAGTCGCAGGAG
**Sertoli Cell**		
*Amh*	TCCTACATCTGGCTGAAGTGATATGGG	AGGTTCTGTGTGCCCCGCAG
*Aqp8*	GCTGGCAGTCACAGTGATCGGA	CCTGGACGATGGCAAAGGCTG
*Ccnd2*	GGAACCTGGCCGCAGTCACC	AATCATCGACGGCGGGTACATG
*Clu*	CCACGCCATGAAGATTCTCCTGC	CTCCCTGGACGGCGTTCTGA
*Cldn11*	TCACAACGTCCACCAATGACTG	GGCACATACAGGAAACCAGATG
*Cst12*	GGATGACGATTTTGCCTACAAGTTCCT	TTCTCTCTCCTGGACCTTCCTGCA
*Cst9*	GATATTTGCCCCTTTCAGGAGAGCC	AGAGAAGTACGTGACCAGTCCATGGG
*Dhh*	GGCGCAGACCGCCTGATG	AAGGCACGGCCTTCGTAGTGG
*Espn*	GCTTCTGGTCGGGCATTACCCT	GTGTCATGCCGTCTTGGGCG
*Fshr*	GGCCAGGTCAACATACCGCTTG	TGCCTTGAAATAGACTTGTTGCAAATTG
*Fyn*	GAAGCGGCCCTGTATGGAAGGTT	TGTGGGCAGGGCATCCTATAGC
*GATA1*	ATGGTCAGAACCGGCCTCTCATC	GAGCTTGAAATAGAGGCCGCAGG
*Il1a*	TTGGTTAAATGACCTGCAACA	GAGCGCTCACGAACAGTTG
*Inhba*	CATGGAGCAGACCTCGGAGATCA	TGGTCCTGGTTCTGTTAGCCTTGG
*Inhbb*	GAGCGCGTCTCCGAGATCATCA	CGTACCTTCCTCCTGCTGCCCTT
*Msi1*	TCACTTTCATGGACCAGGCGG	GTTCACAGACAGCCCCCCCa
*Rhox5*	AGGTTCGCCCAGCATCGACTG	GCCGCAGCCCTCCTGATCTT
*Testin*	AAAGACAATGGCGGCCTCGc	GGCCCCACTTTAGCCACTGCC
*Shbg*	GACATTCCCCAGCCTCATGCA	TGCCTCGGAAGACAGAACCACG
*Spata2*	GCCGTGTGGGCCTGTGCTT	TTCCCCAAATCAAACCCAAGGG
*Sox9*	CGCGGAGCTCAGCAAGACTCTG	TGTCCGTTCTTCACCGACTTCCTC
*Tjp1*	GCGGAGAGAGACAAGATGTCCGC	CTCTGAAAATGAGGATTATCTCTTCCACCA
*Trf*	CAAATGCATCAGCTTCCGTGACC	CGGCATCGTACACCCAACCC
*Wt1*	GCTCCAGCTCAGTGAAATGGACAGAA	GGCCACTCCAGATACACGCCG
*Wnt5a*	CTGCTTCTACCATGCGTTTGCTGG	GCCATGGGACAGTGCGGC
**Germ Cell**		
*Tnp1*	GGCGATGATGCAAGTCGCAA	CCACTCTGATAGGATCTTTGGCTTTTGG
*Spo11*	CGCGTGGCCTCTAGTTCTGAGG	GGTATCATCCGAAGGCCGACAGAAT
*Stra8*	GAAGGTGCATGGTTCACCGTGG	GCTCGATGGCGGGCCTGTG
*cKit*	GCATCACCATCAAAAACGTG	GATAGTCAGCGTCTCCTGGC
*Gfra1*	CAGTTTTCGTCTGCTGAGGTTG	TCTGCTCAAAGTGGCTCCAT
*Vasa*	AGTATTCATGGTGATCGGGAGCAG	GCAACAAGAACTGGGCACTTTCCA
*Boll*	AACCCAACAAGTGGCCCAAGATAC	CTTTGGACACTCCAGCTCTGTCAT
*Crem*	TTCTTTCACGAAGACCCTCA	TGTTAGGTGGTGTCCCTTCT
*Prm1*	TCCATCAAAACTCCTGCGTGA	AGGTGGCATTGTTCCTTAGCA
*Acrosin*	TGTCCGTGGTTGCCAAGGATAACA	AATCCGGGTACCTGCTTGTGAGTT

**Table 2:** Testicular weights and epididymal spermatozoa count in each group at day 120.

Groups	I	II	III	IV	V
Body weight (g)	33.310 ± 0.917	33.905 ± 1.117	26.982 ± 2.103 ^a^	29.470 ± 2.408 ^a^	31.114 ± 1.324 ^a,b^
Testis weight (g)	0.099 ± 0.012	0.100 ± 0.003	0.014 ± 0.002 ^a^	0.035 ± 0.002 ^a,b^	0.100 ± 0.006 ^b^
Epididymal spermatozoa (×10^5^)	20.493 ± 2.362	26.968 ± 1.527 ^a^	0.272 ± 0.011 ^a^	6.673 ± 0.510 ^a,b^	20.893 ± 1.677 ^b^
Fertility rate	100% (10/10)	100% (10/10)	0% (0/10) ^a^	20% (2/10) ^a,b^	100% (10/10) ^b^

Data are presented as mean ± standard deviation. ^a^ *p* < 0.05 vs. group I; ^b^ *p* < 0.05 vs. group III.
